# Highs and Lows of Lambing Time: Sheep Farmers’ Perceptions of the First Outbreak of Schmallenberg Disease in South West England on Their Well-Being

**DOI:** 10.3390/ijerph16245057

**Published:** 2019-12-11

**Authors:** Clare J. Phythian, Mike J. Glover

**Affiliations:** 1 Faculty of Veterinary Medicine, Institute for Production Animal Clinical Science, Norwegian University of Life Sciences (NMBU), 4325 Sandnes, Norway; 2 Bristol Veterinary School, University of Bristol, Langford BS40 4DU, UK; 3 Torch Farm and Equine Ltd., South Molton EX36 4EJ, UK; mikeglover@torchfarmvets.com

**Keywords:** farmer: well-being, sheep, Schmallenberg disease, disease impact, One Welfare

## Abstract

The outbreak of a previously unknown and new disease in the United Kingdom, known as ‘Schmallenberg disease’, a disease associated with abortions, stillbirths and fetal deformities in naïve ewes, was reported for the first time in South West England during the 2012/13 early lambing season. Epidemiological studies confirmed that the Schmallenberg virus (SBV) had a severe negative impact upon animal welfare and the productivity of affected flocks. By contrast, there was a specific lack of research on the impact of SBV on sheep farmer well-being. This study aimed to improve our understanding of sheep farmers’ experiences of Schmallenberg disease, and the impact of the first outbreak on sheep farmer well-being during the 2012/13 early lambing season in South West England. Face-to-face, semi-structured interviews with six farmers with small flocks of pedigree and purebred sheep in South West England were conducted in 2013. The data were analysed via thematic analysis. The main themes regarding the impact of disease on farmer well-being included: (i) emotional highs and lows are part of a normal lambing season; (ii) negative emotions and memories associated with the Schmallenberg disease outbreak; and (iii) resilience and coping with the unexpected disease outbreak. These novel data present preliminary findings from a small number of sheep farmers, and indicate that for some farmers, an unexpected outbreak of a new and emerging disease for the first time during lambing, and dealing with high levels of dystocia, deformities and deaths in their animals, had a negative impact on their emotional well-being during the peak period of the sheep production cycle.

## 1. Introduction

Farmer well-being is a public health concern [1,2]. Well-being encompasses good mental health, as well as wider positive and negative aspects, including a person’s subjective impression of their life, and objective factors, such as financial security and family support [3]. Livestock farming is a stressful occupation, with an ever-shifting economic climate and changing environmental conditions, combined with unpredictable animal health and behavior, long working hours and the highly physical nature of farming work [3,4]. High psychosocial demands with respect to a hard work and production ethos, economic influences and social and environmental responsibility [5,6], create a unique context where both farmer injury and stress are not uncommon [3,4]. Stressors can vary across different farming sectors. For example, financial instability is more stressful for United Kingdom (UK) sheep and beef producers, compared to dairy or arable farmers in the UK who tend to be more stressed by time pressures [7]. The livestock farming business, farmstead and family life are also often inextricably linked, adding to the complexities of responsibilities and relationships [3,8].

One Welfare research into understanding farmer well-being extends, complements and empowers the more clinically-focused One Health approach to interconnect animal welfare, human well-being and environment concerns [9]. The association between farmer well-being and farm animal welfare is well-recognized [10]. Most One Welfare research to-date has focused on how the psychological and emotional well-being of farmers can affect sheep welfare, and particularly, neglectful care [10], or the effect of farmer attitudes and behavior on fear and stress behaviors associated with animal handling [11]. Indeed, recent work confirms that positive farmer attitudes to routine farm management tasks and enjoyment of working with sheep are associated with positive welfare outcomes for the sheep [12]. Conversely, in this study, we were interested in how a disease with severe negative consequences for sheep health and welfare, and specifically the outbreak of a new disease, could have impacted the emotional well-being of sheep farmers.

Lambing time is a very busy, physically demanding period, and is typically associated with even longer and more unsociable working hours than other periods, with farmers taking rapid life–death decisions and emergency actions on top of routine management and care. In 2011, a novel *Orthobunyavirus* associated with the birth of lambs with severe congenital deformities was identified in Northern Europe, and later named as Schmallenberg virus (SBV) [13,14]. Further investigations identified that infection of naïve ruminants with SBV causes abortions, stillbirths and diverse fetal deformities, and abnormalities of the central nervous system [15]. SBV spread rapidly from Germany and the Netherlands, to Great Britain and France and further across the European region [16,17]. SBV is transmitted by arthropod vectors, primarily by biting midges (*Culicoides* spp.) and in temperate regions, a seasonal pattern of spread in summer and autumn periods is reported [18]. Schmallenberg disease was first detected in Great Britain in January 2012 [13]. In South West England, Schmallenberg disease outbreaks were first observed in early lambing sheep flocks during November and December 2012 [19]. Post-outbreak, serological surveys of sheep in this region [20], and later national studies [21,22], indicated that Schmallenberg disease had severe impacts on sheep welfare, reproductive performance and the financial productivity of some affected flocks.

Previous research on the occupational stress of a variety of farmers in South West England was based on a quantitative postal survey, and identified that a significant proportion of respondents had high levels of anxiety and depression [8]. Post-outbreak impact studies of foot-and-mouth disease (FMD) in the UK highlighted the specific lack of emotional support for farmers, who were more likely to turn to their own communities, and to veterinary surgeons, than to mental health professionals [23,24]. Practicing veterinarians often have a good and long-established relationship with farmers, might readily identify concerns for farmer well-being and are often aware of specific animal health and welfare issues on particular farms [10].

Veterinary epidemiological studies of the 2011/2012 Schmallenberg disease outbreak used a national questionnaire to examine the association between quantitative flock welfare measurements, financial outcomes and quantitative emotional well-being scores [21]. However, a simple quantitative well-being score does not reflect the complexity of emotions, and cannot capture in-depth data on the perceived and lived experiences of respondents. We identified a lack of knowledge on the experiences and perceptions of sheep farmers at lambing time, and specifically a lack of understanding of the unexpected nature and impacts of Schmallenberg disease on the emotional and occupational well-being of sheep farmers.

Qualitative research aims to explore interpret and make sense of subject matter and understand the meanings of participants in their natural settings [25]. In other healthcare sectors, interviews and focus groups are common methods of qualitative data capture [26]. Interviews allow participants to provide deep insights, and are more appropriate when addressing sensitive topics as compared to focus groups.

The authors worked with a considerable number of sheep farming clients of a farm animal veterinary practice in South West England. Early lambing flocks of pedigree and purebred sheep were amongst the first in our region to be affected by Schmallenberg disease in November and December 2012. By the summer of 2012, the UK Government had described Schmallenberg disease as a “low-impact disease” [27]. However, by the end of December 2012, this description did not fit with our veterinary experiences in the South West of England. This was supported by data on disease outbreaks in the Netherlands, which identified a considerably higher number of deformed lambs on early lambing flocks compared to spring lambing flocks [28]. However, the impact of higher-than-normal levels of animal death and deformities on farmer well-being had not been explored.

We reflected that as farm animal veterinarians, we were uniquely placed to regularly access and communicate with sheep farmers located in rural areas regarding their personal experiences of Schmallenberg disease. We wanted to better understand their experience of Schmallenberg disease, and to present their untold narratives. This article presents a small, veterinary practice-based research study that aimed to generate insights on the impacts of this novel disease on the occupational well-being of small-scale sheep farmers who were amongst the first farms in South West England to experience Schmallenberg disease during 2012/13.

## 2. Materials and Methods

Purposive sampling was used to recruit participants in South West England. All participants were sheep farming clients of Torch Farm and Equine Ltd., or of South West Sheep Breeding Services, which is a subsidiary of Torch Farm and Equine Ltd. in Devon, England. These flocks employed artificial insemination breeding technologies and/or practiced natural mating using specific purebred or pedigree breeds of sheep to facilitate an early lambing season (November–December), as compared to a more traditional spring-lambing period. We specifically selected flocks with an early lambing period in November and December 2012, since this coincided with the first detection of SBV in South West England [19]. Prior serological sampling by one of the authors identified that these small-scale farms experienced a high exposure of naïve ewes to SBV [20], and were considered to be amongst the first sheep farms in the region to be affected.

Accordingly, farmers of seven small early lambing sheep flocks with clinically confirmed cases of Schmallenberg-affected lambs, who also previously participated in a serological SBV survey [19,20] and who maintained detailed lambing records, were approached for this study. Farmers were invited to participate through contact with one of the authors (MJG) who was the regular veterinary surgeon providing specialized ovine artificial reproductive services. Each farm was invited to participate in a short interview conducted at a time and a location of their choice. All participation was voluntary. Prior to undertaking the study, interview questions were pilot tested with one commercial sheep farmer of Torch Farm and Equine Ltd. to ensure that the interview would take approximately 30 min. Interviews with each of the farmers were conducted during August to October, 2013. Before each interview, the informed, written consent of each participant was obtained.

Background data were collected on participant demographics, farm type, purpose, flock size and breed. During the semi-structured, face-to-face interviews, farmers were asked some open questions on their perceptions and recollections of the first Schmallenberg disease outbreak on their well-being during the November-December 2012 lambing period. All interviews took place around the farmhouse kitchen table, which is a place familiar to UK livestock veterinarians as a space for discussions, reflecting ideas and analyzing farm records. The interviews were led by a veterinary researcher (CJP) that was unfamiliar to the participants and had designed and conducted the interviews in order to capture useful data, and ensured the conversation avoided diverging into a more clinical veterinary or advisory role [26]. The familiar veterinarian (MJG) who had previously collected blood samples to confirm SBV exposure [20] was present to put respondents at ease and build up confidence and rapport around discussing a potentially sensitive topic with an unfamiliar visitor to their farm.

Qualitative data were captured as individual digital voice recordings. The audio recordings were transcribed verbatim and transcripts double-checked against the audio recording for accuracy. Transcripts were analyzed using thematic analyses [29]. Farmer responses were examined and manually coded by one researcher (CJP) to identify broad themes. Coding was later checked by a second researcher (MJG). Where any discrepancy was identified, the descriptions were reviewed and a consensus reached between the two authors.

The study was conducted in accordance with the Declaration of Helsinki, and the protocol was approved in 2013 by the University of Bristol, Faculty of Medical and Veterinary Science Research Ethics Committee (number 3283).

## 3. Results

### 3.1. Farm Demographics

One of the farmers that previously participated in serological SBV sampling declined to participate. Interviews were conducted with the remaining six sheep farmers in South West England during 1^st^ August to 30th September 2013. All participants were of white British ethnicity, including three male and three female respondents, and all were aged between 30 to 60 years. The number of ewes mated in the early breeding season varied from 30 breeding ewes to 320; these data were categorized to maintain participant anonymity (Table 1). All study farms reared pedigree or purebred sheep breeds, including Charollais and Polled Dorset. To maintain anonymity, flock location and breed are not presented together here.

### 3.2. Signs of Schmallenberg Disease Reported by Participating Farmers

Common signs of Schmallenberg disease identified by all or at least 5 out of 6 the sheep farmers included: arthrogryposis (twisted limbs), torticollis, scoliosis or kyphosis of the spine (curved back), brachy/tachgnathia (undershot/overshot jaw), deformed head and stillborn lambs. As well as stillborn lambs, a proportion of severely-affected lambs were alive at birth.

### 3.3. Farmer Well-Being

We identified three main themes related to impact of Schmallenberg disease on farmer well-being.

#### 3.3.1. Emotional Highs and Lows are Part of a Normal Lambing Season

Farmers looked forward to lambing time and the arrival of lots of new life on the farm. Whilst it is a busy and intensive period of work, it is usually an enjoyable time, and these sheep farmers usually experienced job satisfaction and a sense of professional pride. For example: “when things are going great, there’s no better thing to be doing than lambing really” (Farmer B). These farmers accepted that a small number of ewes and lambs might die during the lambing season, usually associated with the birthing process, in spite of their best efforts, and were familiar with the cycle of life, death, disease and the occasional deformities that can arise during even a normal (Schmallenberg disease-free) lambing season. 

For example, one participant said, “To me that’s breeding, that’s what we do, they live or they don’t live…” (Farmer C). When discussing how they coped with the birth of dead lambs, or deaths of neonatal lambs in the lambing time, Farmer F revealed: “you bite the bullet, you just get on with it”. Whilst the birth of congenitally deformed lambs appeared to be relatively rare, it was not an unknown occurrence, and they were familiar with the occasional birth of deformed lambs: “we’ve all seen the odd one, it’s just a whole heap of them at the same time. You always get one.” (Farmer C).

#### 3.3.2. Negative Emotions and Memories Associated with the Schmallenberg Disease Outbreak

The early lambing season of 2012/13 was unlike anything these farmers had ever experienced before. Participants were surprised and shocked by the sudden nature of the outbreak, and unfamiliar high level of dystocia in ewes, the birth of numerous deformed lambs and higher level of ewe and lamb deaths than in previous lambing periods. Farmers recounted that they had experienced a variety of negative emotions, and held on to some lasting unpleasant memories of that lambing period. Dealing with affected ewes and lambs and even the regular routines and lambing activities during the Schmallenberg disease outbreak, was all associated with more negative emotions than experienced in previous lambing seasons. They recalled particular feelings of sadness, heightened stress, anxiety and some also mentioned a feeling stigma and shame from the extent of deaths and deformities they witnessed in their flocks. For example:

Feeling sad: “I was just upset, just upset about the losses isn’t it… any losses are, you know... especially when it’s the ewes. I think lambs... you lose lambs don’t you, but when it comes to the ewes you kind of feel a bit more touched.” (Farmer A).

Feeling stressed: “I think I just remember feeling a little bit more stressed than normal. That’s how it works when you’re under a little bit of pressure, I mean normally you cope with things better at that time.” (Farmer D).

Feeling miserable: “…compared to where we were in 2013 you realize how miserable it was looking back to 2012…in hindsight it was quite a miserable time…” (Farmer B)

Sense of fear and dread for routine work activities: “It affects your mental health. It was difficult, you didn’t want to.. but you just knew you had to go outside (sic. into the lambing shed) again” (Farmer F).

Negative expectations: Farmer E described an expectation of witnessing even more deaths, deformities and dystocia in entering the lambing shed: “It was very depressing at the end, because you knew every time, virtually, you would have a problem.”

Stigma and shame: “I did have this thing that I didn’t really want anyone to see it (dead ewes or deformed lambs)... just shove it in the back” (Farmer B).

Feeling more physically and mentally tired than usual: Whilst farmers had discussed the additional care and support of lambs and ewes only one farmer specifically reflected on feeling more tired, “I had to spend more time with the sheep…sleep took a bit of a backseat, … I was tired.”(Farmer D).

Feeling a lack of control and helpless to treat affected animals: “Well I don’t like things like that, I just… I’m not very good, I don’t like hospitals, I don’t like veterinary practices, when you are out of control and you can’t do anything for that animal, you feel out of control, it’s a bit crap really.. I didn’t like it very much.” (Farmer C).

#### 3.3.3. Resilience and Coping with the Unexpected Disease Outbreak

Whilst the unexpected, higher level of dystocia, deformities and death of livestock than usual was recognized to be a mental and physical stressor for most participants, many farmers also shared feelings of resilience and being able to cope. Half of these sheep farmers expressed gratitude for their situation and empathized that the outbreak “could have been worse”. They also expressed gratitude and relief that Schmallenberg disease was not zoonotic (e.g., “I was just thinking it’s not in my children, thank goodness it’s not in my children.” [Farmer C]). It appeared that they were able to cope with a one-off, isolated outbreak. For example, one participant said, “it affected me a little bit, but not, not so bad, if it was going to happen every year, I wouldn’t do it again.” (Farmer C). 

Farmers also expressed empathy for other farmers who were in a worse situation than themselves: “speaking to friends and neighbors they had something much worse than we had” (Farmer B). Similarly, Farmer F recalled: “I was fearful then that we were in trouble, but thankfully we weren’t in as much trouble as some people, or didn’t experience as much trouble”. Whilst Farmer D reflected: “from a positive point of view when things go well I think I appreciate it a bit more now than then, looking on the bright side really.”

## 4. Discussion

To our knowledge, this is the first time the emotional well-being of sheep farmers that experienced an unexpected outbreak of an emerging animal disease at lambing time has been qualitatively explored. The Schmallenberg disease outbreak was a shock and an additional source of strain and stress amongst the usual routines and experiences of the lambing season.

Lambing time is usually a source of emotional “highs” associated with joy and a sense of pride in the successful delivery of new life onto the farm and a job well done. However, these farmers also shared recollections and memories of the many more “lows” experienced that year due to the unexpected nature, severity and intensity of death, deformities and dystocia that they observed as a result of Schmallenberg disease, compared to usual lambing seasons. Despite the acceptance and familiarity with the cycle of life and death at lambing time, a key finding was that the intensity of issues and experiences of the November-December 2012 early lambing period held very negative memories for some farmers, even one year after the disease outbreak.

The occurrence of a previously unknown and unexpected novel disease, that had severe implications on animal welfare and increased ewe and lamb mortality rates, resulted in increased farmer workload and physical activities. We found no studies specific to farmers, but lack of restful sleep, and changes in sleep patterns from working long shifts and unsocial hours, are known risks for social and mood disorders, as well as an array of chronic health conditions [30].

Most farmers reported giving more physical assistance and lambing intervention, with a higher number of dystocia cases in ewes. Farmers spent more time and effort than normal in supporting affected lambs to suckle, and they perceived much greater physical and mental strain than in other lambing periods. These preliminary findings suggested that an unexpected and novel disease occurring at the very time that farmers usually look forward to is more impactful on their well-being than other events and causes of animal deaths in the production cycle.

The entry of new life on the farm is not without risk, and it is well-known that the highest risk of ewe and lamb mortalities are associated with the peri- and immediate post-lambing period [31]. Whilst the flock-level prevalence of congenital deformities in normal lambing seasons is generally low, it is not an unknown phenomenon in sheep. However, the level of deformities witnessed in both live and stillborn lambs affected by Schmallenberg disease is something that had not been previously reported [15]. As elsewhere, the most frequent signs observed by farmers were arthrogryposis and spinal vertebral column deformities (e.g., stiff neck, torticollis, scoliosis, kyphosis) [21,32]. A recollection common to four of these farmers was the sense of dread in returning to the lambing shed, possibly associated with anxiety and fear following their first-hand experiences of the first cases of Schmallenberg disease in their lambing ewes.

A new insight was identifying some reports of shame and self-stigma around the losses in the lambing shed and concealment from family or visitors expressed by some participants. High levels of personal stigma, often presenting as feelings of shame and guilt, are recognized as very harmful to personal mental and physical well-being [33]. However, it was not until lambing time that the disease was first detected. The emergence of Schmallenberg disease was impossible for these producers to have foreseen, and difficult, in hindsight, to have prevented.

The emotional “lows” from witnessing Schmallenberg disease in lambing ewes and newborn lambs, and additional work created by the outbreak, led to feelings of stress, sadness, dread and misery, and experienced feeling more physically tired as compared to prior Schmallenberg disease-free lambing seasons. 

Whilst this research study is relatively small in scale, some familiar themes of stress, anxiety, depression and psychological distress are highlighted here, which concur with One Welfare concerns for farmer mental health identified by larger studies of livestock farmers using validated psychometric scale testing [2,34].

Our findings add to earlier epidemiological surveys examining the impact of SBV on the 2011/2012 [21] and 2016/2017 lambing periods in farms within Great Britain [35], which identified that many farmers scored Schmallenberg disease as having a high emotional impact. However, half of these participants also described features of resilience, such as the ability to ‘thrive in the face of adversity’ [2]. In spite of the challenges, half of these participants expressed gratitude for their situations and empathy for others. These farmers reflected that things “could have been worse” during the outbreak; relating back to personal health issues, the well-being of their families and the non-zoonotic nature of SBV infection. All of the study participants have remained in sheep farming, with the same breeds, and are still practicing early-lambing seasons since the 2012 outbreak of Schmallenberg disease. Other studies identified that a number of sheep farmers reported they would be less likely to farm sheep in future because of the SBV experience in 2011/2012 [21] or the later 2016/2017 outbreak [35]. Only one participant indicated considering leaving sheep farming if all future lambing seasons were as traumatic as in 2012.

### Study Limitations

Limitations of this study include the small sample size and a sampling approach that focused on small early lambing flocks of pedigree and purebred sheep. While we were limited by finances and resources, we believe that this approach was valid as a first-step towards a better understanding of the impact of a Schmallenberg disease outbreak on farmer well-being. We present these findings as novel, preliminary data on the subject and a starting point for wider studies on understanding sheep farmer well-being, particularly around lambing time. Another limitation is that we did not ascertain the meaning of “well-being” to these farmers, as recently reported by Crimes and Enticott in their study of the impact of an endemic livestock disease [36]. Whilst we identified some diverging perceptions around the impact and reflections of SBV, the small sample size likely precluded data saturation [37].

All interviews were conducted approximately one year following the initial SBV outbreak. Farmer responses were, therefore, open to medium to long-term recall bias [38]. Previous studies of livestock farmers in South West England identified higher stress scores in women respondents [8]. However, there were insufficient data in the present study to explore potential gender-related differences. In addition to extending the geographical area and number of participants, it would also be useful to capture the perceptions and impact of unexpected animal disease, death and deformities on the well-being of other farm workers, including experienced shepherds who are employed as additional labor, as well as veterinary and agricultural students who often undertake unpaid lambing placements.

## 5. Conclusions

Lambing can be an exciting, exhilarating, exhausting and emotional period for sheep farmers, who can face a number of multifaceted psychosocial demands, expectations and stressors associated with this peak and concentrated period of activity. This novel research highlights impacts of a completely new and unexpected livestock disease outbreak on the occupational well-being of sheep farmers who were amongst some of the first farmers in South West England to witness Schmallenberg disease. This work also highlights the value of including practicing veterinarians in accessing and communicating with regional sheep farmers on One Welfare topics pertinent to farmers, as well as animal health and welfare. Further research is needed to explore the emotional well-being of a wider population of sheep farmers, examine the impacts of emerging, exotic and endemic diseases or other crises and identify any needs for support or interventions that may be specific to sheep farmers, particularly around lambing time.

## Figures and Tables

**Table 1:** Demographic characteristics of participating sheep farmers.

Participant Code	Ethnicity	Gender	N Ewes Mated
A	White, British	Female	25–50
B	White, British	Female	100–150
C	White, British	Female	100–150
D	White, British	Male	>300
E	White, British	Male	25–50
F	White, British	Male	25–50

N = abbreviation for Number.
